# Oxidative Stress and Oocyte Developmental Competence: A Cell-Specific Analysis of Antioxidant Enzymes in Human Follicle Cells

**DOI:** 10.3390/medicina62091734

**Published:** 2026-09-09

**Authors:** Giovanni Ruvolo, Gianna Rossi, Daniele Lozzi, Gerlando Cocchiara, Beatrice Ermini, Marta Scarcella, Michele Ermini, Ettore Cittadini, Sandra Cecconi

**Affiliations:** 1 Centro Italiano di Procreazione Assistita-Studio Diagnosi Medica, Viale Regina Margherita 270, 00198 Roma, Italy; 2 Centro di Biologia Della Riproduzione S.r.L., Via Valerio Villareale 54, 90141 Palermo, Italy; 3 Department of Life, Health and Environmental Sciences, University of L’Aquila, 67100 L’Aquila, Italy; 4 Medicina di Precisione in Area Medica, Chirurgica e Critica, University of Palermo, Via Liborio Giuffrè 5, 90127 Palermo, Italy

**Keywords:** antral follicle diameter, cumulus cells, granulosa cells, oxidative stress, ongoing pregnancy rate

## Abstract

*Background and Objectives*: Granulosa (GCs) and cumulus cells (CCs) play a key supportive role in oocyte developmental competence and in antioxidant defence mechanisms triggered by endogenous reactive oxygen species production. Assisted reproductive technology (ART) procedures are an additional source of oxidative stress (OS) that somatic cells must counteract to ensure healthy embryo production. In this study, we quantified the major antioxidant enzymes (CAT, GPx1, SOD-1 and 2) in GCs and CCs retrieved from human antral follicles classified as large (>18 mm, L) or small (<18 mm, S) using ELISA. We correlated their contents with ongoing pregnancy rate (OPR). *Materials and Methods*: Healthy women (n = 17) undergoing ART procedures were selected for this study. During pickup, antral follicles were classified by diameter as L or S, and their somatic cells were stored separately for ELISA. After intracytoplasmic sperm injection (ICSI), embryo quality was assessed morphologically, and the OPR was evaluated. *Results*: Results showed that embryo quality and OPR were positively correlated with higher antioxidant enzyme levels detected in both GCs and CCs independently of follicle size. *Conclusions*: The data suggest that adequate levels of antioxidant enzymes in GCs and CCs are necessary not only to protect the oocyte from oxidative damage, but may also help predict embryo quality and OPR.

## 1. Introduction

In recent years, attention has increasingly focused on the follicular microenvironment as a determinant of oocyte developmental competence. The somatic cells of the ovarian follicle, granulosa (GCs) and cumulus cells (CCs), provide the oocyte with the proper microenvironment and signals during growth, maturation, and fertilization [1,2]. In turn, the dynamic interaction among GCs, CCs, and follicular fluid (FF) regulates follicular redox balance, steroidogenesis, and metabolism through various bioactive factors that support oocyte quality [3,4]. Ovarian somatic cells are continuously exposed to reactive oxygen species (ROS), whose excessive accumulation can induce oxidative stress (OS), cellular damage, and apoptosis, finally compromising oocyte and embryo quality [3,5,6]. Therefore, the antioxidant defence system in GCs and CCs is crucial for neutralizing ROS and preserving follicle physiology. Key enzymes such as catalase (CAT), glutathione peroxidase 1 (GPx1), superoxide dismutases (SOD-1 and SOD-2), and glutathione S-transferase (GST) work collaboratively to mitigate OS [7,8]. Recent studies have highlighted the role of these antioxidant molecules in preserving follicle quality and in predicting ART outcomes [9,10]. In fact, ROS-induced oxidative damage has been linked not only to age but also to infertility and even to ovarian stimulation protocols. GCs from older women widely show reduced expression of SOD-1, SOD-2, and CAT, along with mitochondrial defects and increased susceptibility to apoptosis [10,11]. Additionally, CCs from high responders show elevated GST activity and low malondialdehyde (MDA) levels, reflecting a more robust antioxidant defence than in poor and normo-responders [12]. Also, CCs from patients with moderate/severe endometriosis exhibit increased SOD-1 expression, which correlates positively with pregnancy outcomes following ICSI [13]. The critical role of antioxidants in mitigating OS is well evidenced by the finding that treatment of human luteinized GCs with exogenous Cu^2+^/Zn^2+^-SOD (200 U/mL) efficiently reduces caspase-3 activity and preserves cellular viability [14]. Therefore, investigating the expression and activity of antioxidant enzymes in CCs and GCs helps clarify the mechanisms orchestrating follicular maturation and fertilization success. In this observational study, we further investigated the association between antioxidant enzyme levels in ovarian somatic cells and the OPR. To this end, we sorted preovulatory follicles into two groups: large (L, >18 mm) or small (S, <18 mm). Levels of CAT, GPx1, SOD-1 and SOD-2 were determined in GCs and CCs retrieved from the two different follicle classes to evaluate if these levels could affect embryo quality and OPR.

## 2. Materials and Methods

### 2.1. Chemicals

Chemicals used in this study were obtained from the following companies: rabbit monoclonal anti-CAT (MAB-94599), SOD-1 (MAB-94600), SOD-2 (MAB-94601) and mouse monoclonal GPx1 (MAB-94602) from Immunological Sciences (Rome, Italy); secondary goat anti-mouse IgG conjugated to HRP (sc-2005) from Santa Cruz Biotechnology (Santa Cruz, CA, USA); and goat anti-rabbit IgG conjugated to horseradish peroxidase (HRP) (cat. 111-035-003) from Thermo Fisher Scientific (Waltham, MA, USA). All of the other reagents and ABTS (2,20-azinobis (3-ethylbenzothiazoline-6-sulfonic acid)-diammonium salt) were purchased from Sigma (St. Louis, MO, USA) and were of the purest analytical grade.

### 2.2. Selection of Patients and Follicular Classification

The study was conducted at the ART clinic CIPA-SDM in Rome, Italy, between May and December 2022. The study enrolled 17 women with similar age (35 ± 2.5 years) undergoing ART cycles with ICSI. The local Institutional Ethics Committee approved the protocol, and all participants provided written informed consent before inclusion, in compliance with the Declaration of Helsinki. Patients were accurately selected using criteria that excluded those with endometriosis, heavy smokers, poor responders, maternal age ≥ 38 years, BMI > 24 or <18, and alcohol and drug abuse. Regarding ovarian stimulation, the mean total doses of recombinant FSH and LH administered to all selected patients were 2366 ± 865 IU and 1803 ± 200 IU, respectively. Mean estradiol (E2) value on the day of ovulation triggering was 2524 ± 805 pg/mL.

During follicular aspiration, each follicle’s diameter was measured in each patient, and follicles were classified as small (S) (diameter < 18 mm) or large (L) (diameter ≥ 18 mm). Cumulus–oocyte complexes retrieved from the S or L follicles were separated and denuded from surrounding CC using a combination of enzymatic (80 IU/mL hyaluronidase) and mechanical pipetting under a stereomicroscope. Only metaphase II (MII) oocytes, identified by the presence of the first polar body, were utilized for ICSI. Oocytes from S or L follicles were incubated separately according to follicle size in culture medium (G-TL, Vitrolife, Gothenburg, Sweden) at 37 °C in an atmosphere of 6% CO_2_ and 5% O_2_ until microinjection. Spermatozoa were prepared using the swim-up technique. Motile, morphologically normal sperm were selected under an inverted microscope equipped with Hoffman Modulation Contrast optics (Modulation Optics, Inc., New York, NY, USA) at ×400 magnification.

ICSI was performed using a micromanipulation system consisting of an inverted microscope (Nikon, Tokyo, Japan) equipped with micromanipulators and microinjectors (Narishige, Tokyo, Japan). Oocytes were individually injected and then incubated in G-TL culture medium under standard conditions (37 °C, 6% CO_2_, 5% O_2_). Fertilization was assessed about 16–18 h later and confirmed by the presence of two pronuclei (2PN) and two polar bodies; zygotes were further cultured to the blastocyst stage. We then split patients into two groups: those who achieved a clinical pregnancy and those who did not. Ongoing pregnancy rate (OPR) was defined as a viable intrauterine pregnancy with fetal cardiac activity beyond 12 weeks of gestation, and it was calculated as the proportion of embryo transfer cycles resulting in an ongoing pregnancy.

### 2.3. Cumulus and Granulosa Cell Collection and Analysis

After oocyte removal, CCs were washed in a buffered medium MOPS (Vitrolife, Sweden) and centrifuged at 300× *g* for 7 min, and the pellet was resuspended in 30 µL RIPA buffer containing protease inhibitors. Also, GCs recovered from the FF of S or L follicles were pooled, washed in MOPS, and centrifuged at 300× *g* for 7 min. After discarding the supernatant, the pellet was resuspended in 30 µL RIPA buffer containing protease inhibitors. All the samples were stored at −80 °C until processing.

### 2.4. Elisa Assay

We assessed the expression levels of Catalase (CAT), glutathione peroxidase 1 (GPx1), Superoxide Dismutase 1 (SOD-1), Superoxide Dismutase 2 (SOD-2) using enzyme-linked immunosorbent assay (ELISA), as previously reported [15]. Briefly, GC or CC lysates (5 μg/well) were added to coating buffer (0.05 M Na_2_CO_3_, pH 9.6) and incubated at room temperature for 2 h. Samples were incubated for 1 h in 1% bovine serum albumin (BSA) in phosphate-buffered saline (PBS) and then for 2 h in the presence of anti-CAT (1:2000), anti-SOD-1 (1:2000), anti-SOD-2 (1:2000) and anti-GPx1 (1:2000) antibodies, all diluted in 0.1% BSA + 0.025% Tween-20 in PBS. After rinsing 3 times in PBS-Tween 20, 100 μL of anti-rabbit IgG (HRP) or anti-mouse IgG (HRP), both diluted 1:5000 in 0.1% BSA + 0.025% Tween-20 in PBS, was added, and the ELISA plate was incubated for 1 h at room temperature. Finally, the HRP enzyme activity was determined by adding ABTS (100 μL), followed by 1% SDS (100 μL) to stop the reaction. We used a Multiskan ELISA Microplate Reader (ThermoLabsystems, Beverly, MA, USA) to measure absorbance at 405 nm. We determined the ELISA linearity ranges for the antibodies used by dose–response curves with different amounts (0, 2.5, 5.0, 10 μg/well) of human GCs for each antibody. All data were within these linearity ranges and were expressed as absorbance units.

### 2.5. Statistical Analysis

All experiments were repeated at least three times, and data were expressed as the mean ± SEM. Student’s *t*-test was used for comparison between the experimental groups. Results were considered statistically significant when *p* < 0.05. Correlation analysis aimed to evaluate the relationship between enzyme levels and OPR was performed using the Pearson correlation coefficient (r). All statistical analyses were performed using the statistical package SigmaPlot v.11.0 (Systat Software Inc., San Jose, CA, USA).

## 3. Results

### 3.1. MII Retrieval and ICSI Outcome

A total of 118 MII oocytes were retrieved from 146 aspirated ovarian follicles. MII oocytes were sorted based on the diameters of their respective follicles (S or L), and then further assigned to the groups of Pregnant (P) and Not Pregnant (NP) patients. As shown in Table 1, most of the MII oocytes of P patients were retrieved from L follicles, while in the NP group, a similar number of MII oocytes were obtained from both L and S follicles.

Overall, an average of 3.7 ± 2.6 and 3.6 ± 2.5 MII-stage oocytes from P or NP patients were injected by ICSI. Fertilization rate, assessed by the presence of 2PN, was 85% and 84%, respectively (*p* > 0.05). As shown in Table 2, both groups had a similar number of blastocysts transferred. We transferred only blastocysts classified as high morphological grade according to the Istanbul consensus [16]. In both patient groups, similar numbers of blastocysts were obtained from L follicles (*p* > 0.05; Table 2). In the P group, the OPR was 38.7%, and the implantation rate was 21.4%.

### 3.2. Assessment of Antioxidant Enzyme Levels

**Figure 1:**
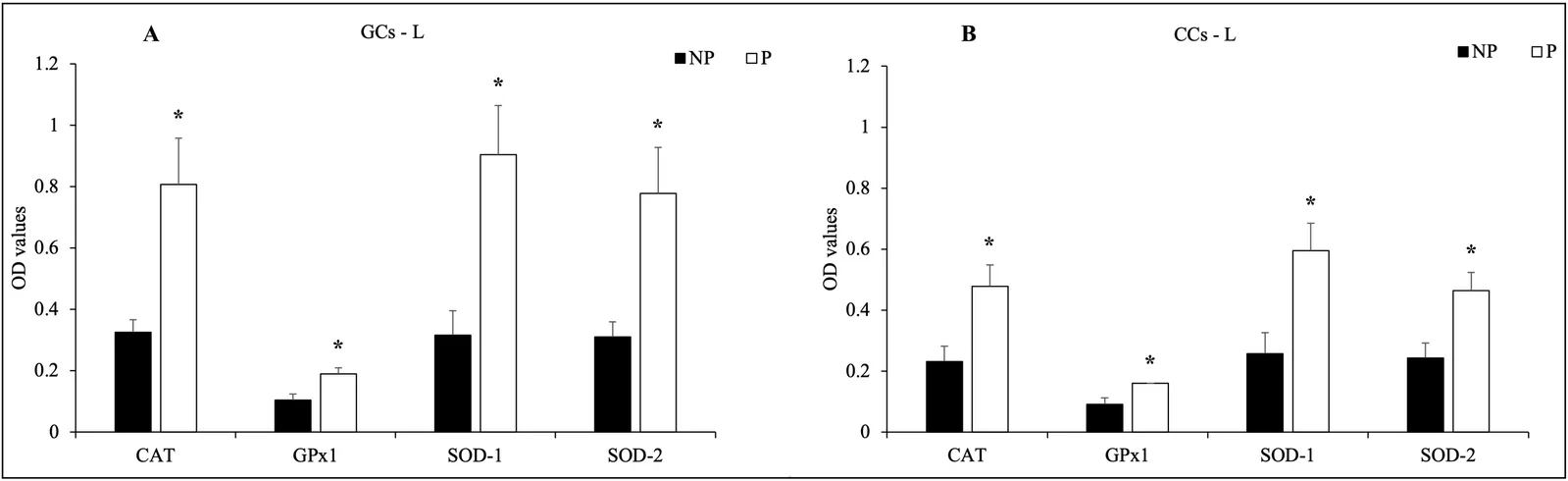
Expression levels of antioxidant enzymes in human GCs (**A**) and CCs (**B**) retrieved from L follicles obtained from NP or P patients. Results of three experiments expressed as mean OD values ± SEM (* *p* < 0.05).

**Figure 2:**
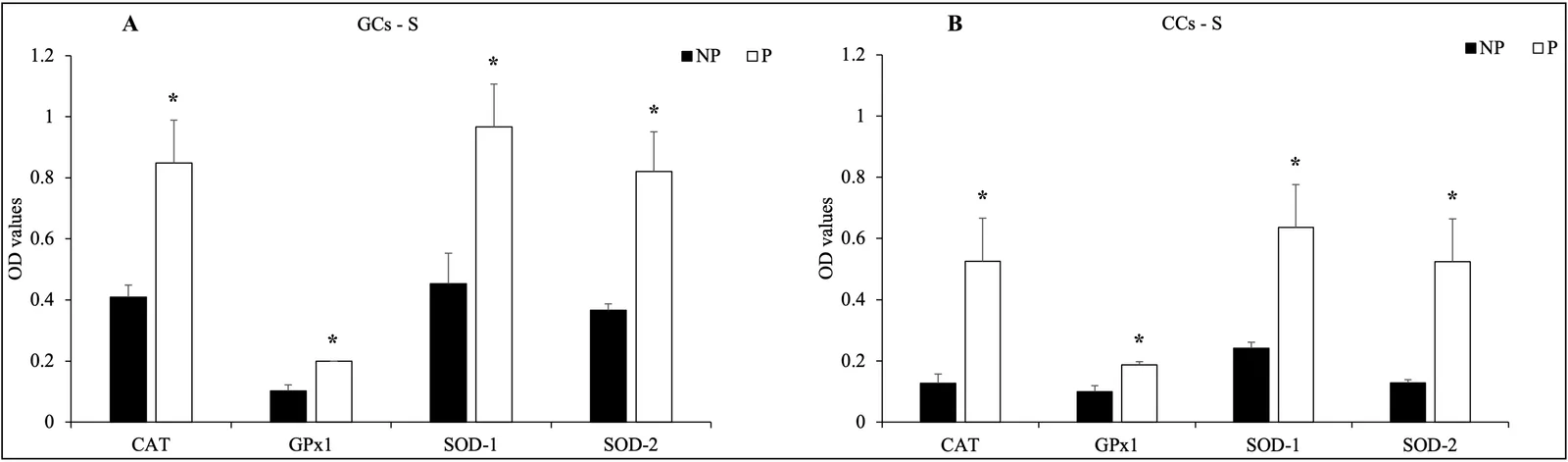
Expression levels of antioxidant enzymes in human GCs (**A**) and CCs (**B**) retrieved from S follicles obtained from NP or P patients. Results of three experiments expressed as mean OD values ± SEM (* *p* < 0.05).

Because biological samples were scarce, we quantified antioxidant enzyme levels in GCs and CCs from L or S follicles by ELISA [15]. Data from P (n = 8) or NP (n = 9) patients were pooled and are reported in Figure 1 for L follicles and in Figure 2 for S follicles.

Experimental results showed that CAT, GPx1, SOD-1, and SOD-2 levels were not significantly different between GCs and CCs obtained from L follicles (Figure 1A,B) or S follicles (Figure 2A,B). However, these levels were significantly higher in the P group than in the NP group (P vs. NP: *p* < 0.05). Our results also indicated that GPx1 was consistently less expressed in both follicle classes (*p* < 0.05).

### 3.3. Correlation Analysis Between Enzyme Levels and OPR

**Figure 3:**
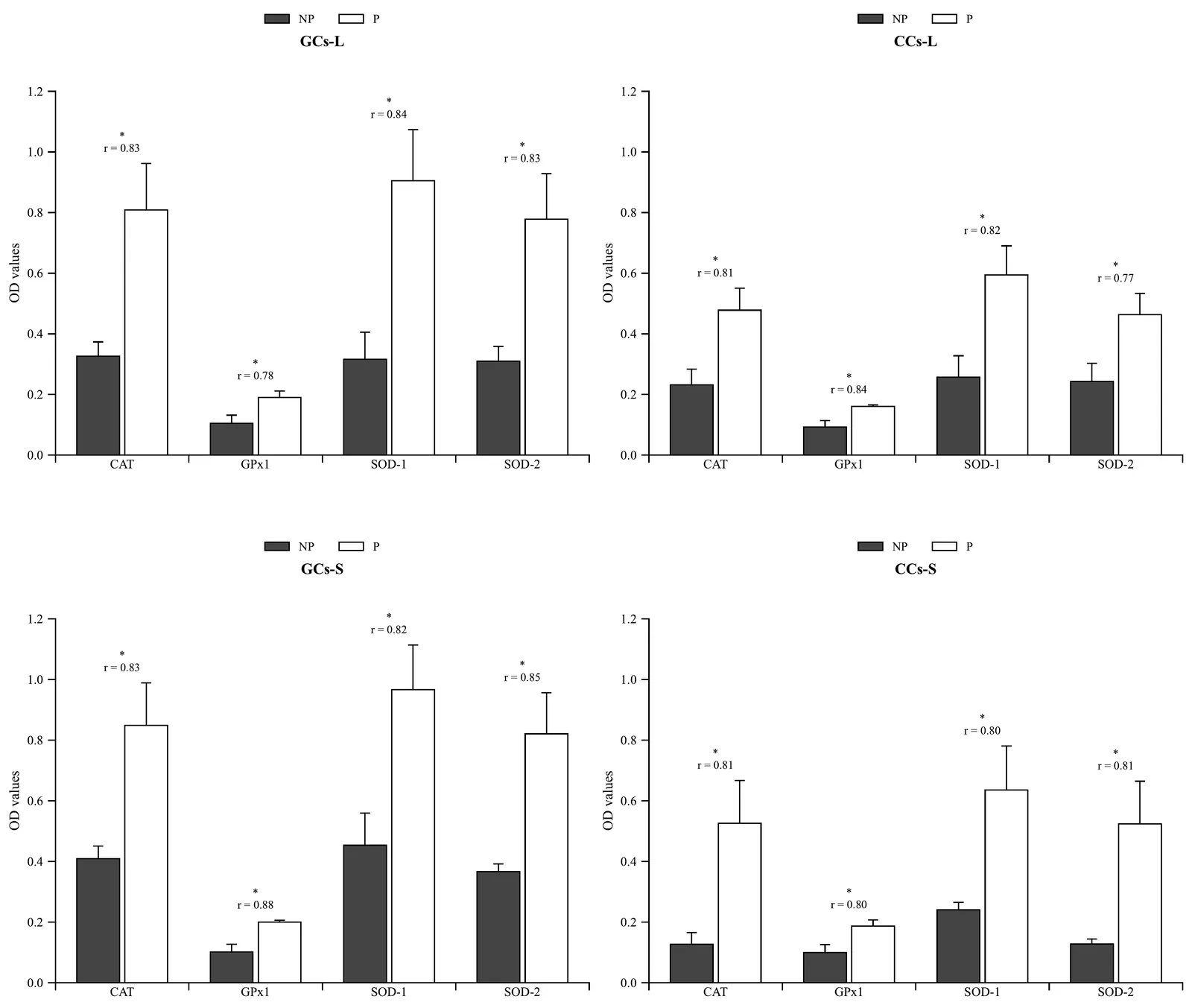
Determination of Pearson correlation coefficient (r) indicates a positive relationship between antioxidant enzyme levels and OPR (* *p* < 0.05).

As shown in Figure 3, a significant positive correlation between antioxidant enzyme levels and OPR was observed (*p* < 0.05).

## 4. Discussion

Somatic follicle cells are a fundamental support system for the oocyte, providing an appropriate antioxidant defence that results in higher oocyte competence and improved ART outcomes. The balance between ROS generation and enzyme antioxidant levels within CCs and GCs is likely critical for oocyte competence, because ROS can act as either signalling molecules necessary for follicular development or mediators of excessive oxidative damage [17,18]. In this prospective observational study, we used ELISA to assess the levels of the principal antioxidant enzymes, i.e., catalase (CAT), superoxide dismutases (SOD-1 and SOD-2), and glutathione peroxidase 1 (GPx1), in granulosa (GCs) and cumulus (CCs) cells derived from the same follicle. We found that these levels were comparable in follicles of different sizes (L or S), but significantly higher in P patients. Notably, P patients have more L than S follicles. Recent reports support a link between robust antioxidant responses in the follicle and improved fertilization and embryo development metrics [18,19]. von Mengden et al. [20] extensively reviewed the association between CC/GC antioxidant markers and oocyte/embryo quality, highlighting that redox activity in human CCs is modulated by age, diagnosis, and even stimulation protocol. Moreover, specific components such as GPx1 and 3, Glutathione, Thioredoxin, and Thioredoxin reductase directly correlate with embryo quality and development. These conclusions highlight that patient-level differences in antioxidant enzyme contents map onto developmental competence.

Donabela et al. [13] reported increased SOD-1 expression in CCs from women with moderate–severe endometriosis. They suggested that this may reflect an adaptive response of CCs to a pro-oxidant follicular milieu. Although it has not yet been determined whether this pathophysiological context reflects a protective or merely reactive upregulation, elevated SOD-1 levels in CCs and GCs may be associated with pregnancy. In other studies [21,22], total SOD activity and other antioxidant measures vary with follicle development stage in complex ways. Indeed, SOD and other antioxidant markers change dynamically during follicular development rather than following a linear trend, and SOD activity may increase or decrease during follicle maturation. Thus, the antioxidant response depends on species, developmental stage, and the biological compartment analyzed. However, animal studies have shown that genetic deficiency of SOD isoforms severely impairs female fertility, highlighting that both insufficient antioxidant defence and disrupted redox homeostasis can adversely affect reproductive function [5,23]. Thus, higher antioxidant response in pregnancy-associated follicles fits a model in which a balanced, sufficiently high antioxidant content is necessary to ensure full oocyte and embryo competence [19].

Our results show that elevated levels of CAT, GPx1, SOD-1 and SOD-2 are similar in both GCs and CCs, and always independent of follicle size but higher in patients achieving an OCP. This may indicate that improved capacity to buffer ROS spikes during the final stages of antral development and ovulation preserves mitochondrial function and genomic integrity in the oocyte and early embryo. This interpretation aligns with studies linking DNA damage and oxidative insults in CCs and GCs to poorer fertilization and embryo outcomes [20]. An alternative explanation is that higher antioxidant protein abundance detected in P patients could be a marker of overall follicular health rather than the sole causative factor. Indeed, follicles with optimal endocrine signalling, mitochondrial fitness, energy metabolism, and a strong antioxidant response support the efficient development of competent oocytes [24]. The physiological burst of ROS triggers ovulation, which activates mechanisms typical of acute inflammation, such as the production of inflammatory molecules and mediators, including prostaglandins and cytokines [25].

An interesting aspect of this work is that GCs and CCs were obtained from the same follicle, thus enabling a more precise within-patient comparison between the two different classes of antral follicles. Prospective collection and clinical follow-up based on OPR assessment could add translational relevance, given the positive correlation between biomarkers and OCP. If validated in larger, independent cohorts, measuring antioxidant enzyme levels in follicle somatic cells could provide noninvasive biomarkers to predict oocyte developmental competence at retrieval. However, antioxidant supplementation strategies have had mixed results in the ART setting, and there is emerging recognition that excessive antioxidant dosing can impair physiological redox signalling necessary for ovulation and early embryo development. Thus, therapies aimed at modulating follicular redox balance should be precisely targeted and based on functional assays rather than on indiscriminate antioxidant use [26,27].

## 5. Conclusions

Our data suggest that higher levels of antioxidant enzymes (CAT, GPx1, SOD-1, SOD-2) detected in GCs and CCs may be associated with positive OPR. These results should be considered exploratory, as we did not perform predictive analyses or independent validation. The study included a relatively small number of patients (n = 17), which may limit statistical power and the generalizability of the clinical findings, particularly given the multifactorial nature of pregnancy outcomes. Larger cohorts and independent validation populations are needed to determine whether antioxidant enzyme levels have clinical predictive value before considering their use as biomarkers of oocyte competence. Given the field’s heterogeneous reports and mechanistic complexity, future studies should quantify oxidative damage and related metabolic pathways at the single-follicle level and investigate whether targeted modulation of the follicular redox environment can safely improve reproductive outcomes.

## Figures and Tables

**Table 1:** MII recovery from L and S follicles obtained from P and NP patients.

Patients (n)	MII from L Follicles Mean ± SD (n. Oocytes)	MII from S Follicles Mean ± SD (n. Oocytes)	Total MII Mean ± SD (n. Oocytes)
P (n = 8)	2.9 ± 1.4 * (n = 50)	0.8 ± 0.6 * (n = 5)	3.7 ± 2.6 (n = 55)
NP (n = 9)	1.9 ± 1.3 (n = 38)	1.7 ± 1.2 (n = 30)	3.6 ± 2.5 (n = 68)

* *p* < 0.05.

**Table 2:** Embryos obtained from S and L follicles of P and NP patients.

Patients	Embryos from L Follicles Mean ± SD	Embryos from S Follicles Mean ± SD	Transferred Embryos Mean ± SD
P (n = 8)	0.9 ± 0.4	0.3 ± 0.2	1.2 ± 0.6
NP (n = 9)	0.5 ± 0.2	0.6 ± 0.6	1.1 ± 0.8

## Data Availability

The datasets generated during and/or analyzed during the current study are available from the corresponding authors on reasonable request.

## Abbreviations

GCs	Granulosa Cells
CCs	Cumulus Cells
ART	Assisted Reproductive Technology
OS	Oxidative Stress
CAT	Catalase
GPx1	Glutathione Peroxidase 1
SOD-1	Superoxide Dismutase 1
SOD-2	Superoxide Dismutase 2
ELISA	Enzyme-Linked Immunosorbent Assay
OPR	Ongoing Pregnancy Rate
ICSI	Intracytoplasmic Sperm Injection
FF	Follicular Fluid
ROS	Reactive Oxygen Species
GST	Glutathione S-Transferase
MDA	Malondialdehyde
L	Large
S	Small
HRP	Horseradish Peroxidase
ABTS	(2,20-azinobis (3-ethylbenzothiazoline-6-sulfonic acid)-diammonium salt)
E2	Estradiol
MII	Metaphase II
2PN	2 Pronuclei
BSA	Bovine Serum Albumin
P	Pregnant
NP	Not Pregnant
OD	Optical density
